# Therapeutic Strategies for Oxidative Stress-Related Cardiovascular Diseases: Removal of Excess Reactive Oxygen Species in Adult Stem Cells

**DOI:** 10.1155/2016/2483163

**Published:** 2016-09-07

**Authors:** Hyunyun Kim, Jisoo Yun, Sang-Mo Kwon

**Affiliations:** Laboratory for Vascular Medicine and Stem Cell Biology, Convergence Stem Cell Research Center, Medical Research Institute, Pusan National University School of Medicine, Yangsan 50612, Republic of Korea

## Abstract

Accumulating evidence indicates that acute and chronic uncontrolled overproduction of oxidative stress-related factors including reactive oxygen species (ROS) causes cardiovascular diseases (CVDs), atherosclerosis, and diabetes. Moreover ROS mediate various signaling pathways underlying vascular inflammation in ischemic tissues. With respect to stem cell-based therapy, several studies clearly indicate that modulating antioxidant production at cellular levels enhances stem/progenitor cell functionalities, including proliferation, long-term survival in ischemic tissues, and complete differentiation of transplanted cells into mature vascular cells. Recently emerging therapeutic strategies involving adult stem cells, including endothelial progenitor cells (EPCs), for treating ischemic CVDs have highlighted the need to control intracellular ROS production, because it critically affects the replicative senescence of* ex vivo* expanded therapeutic cells. Better understanding of the complexity of cellular ROS in stem cell biology might improve cell survival in ischemic tissues and enhance the regenerative potentials of transplanted stem/progenitor cells. In this review, we will discuss the nature and sources of ROS, drug-based therapeutic strategies for scavenging ROS, and EPC based therapeutic strategies for treating oxidative stress-related CVDs. Furthermore, we will discuss whether primed EPCs pretreated with natural ROS-scavenging compounds are crucial and promising therapeutic strategies for vascular repair.

## 1. Introduction

Cardiovascular diseases (CVDs), including ischemic heart disease, stroke, and hypertensive heart diseases are the leading cause of death worldwide [1]. Multiple factors of hemodynamic conditions including shear stress, laminar flow, turbulent flow, extracellular signaling proteins, including interleukins, chemokines, and cytokines, and intracellular biochemical molecules including reactive oxygen species (ROS) affect the condition of blood vessels [2–4]. High blood pressure and inflammatory reaction induced damage of blood vessels lead to hypertension, ischemic heart disease, stroke, and so forth [5, 6]. Several studies have focused on developing drug and stem cell-based therapeutic strategies for repairing ischemic blood vessels and for preserving a healthy and intact blood-endothelial barrier in patients with CVDs [7].

Recent studies have reported that uncontrolled overproduction of oxidative stress-related factors, including ROS, causes CVDs [8], atherosclerosis [9], and diabetes [10]. ROS, which are chemically reactive molecules, a spontaneously produced metabolic by-product in healthy cells, nicotinamide adenine dinucleotide phosphate (NADPH) oxidase, a superoxide-producing enzyme, present in vascular endothelial and adventitial cells, is involved in ROS production [11]. Pathophysiological conditions induce an imbalance between ROS (also known as oxidants) and antioxidants. Excess ROS not only affect blood vessels, but also promote the homing of endothelial progenitor cells (EPCs) into peripheral blood [12, 13]. Accumulating evidences clearly suggest that EPCs recruited to injured ischemic sites induce neovessel formation, leading to the repair of injured tissues.

EPCs were originally identified as angiogenic progenitor cells derived from the bone marrow (BM) and blood [14], as well as other organs or tissues, including cord blood, fetal liver, and skeletal muscles. Circulating EPCs mobilized in response to ischemic repair signaling may directly reach ischemic injury sites and proliferate and differentiate* in situ* into mature endothelial cells (ECs) or smooth muscle cells (SMCs) [15] or may indirectly promote the proliferation or differentiation of resident ECs, resulting in the production of multiple angiogenic cytokines at ischemic sites. Many clinical studies have also reported that EPC dysfunction is closely correlated with vascular homeostasis and various CVDs, such as myocardial infarction, stroke, and hypertension [16]. Accumulating reports recently provide stem/progenitor cell-based therapy strategies using EPCs [7]. The pivotal process of these strategies can be simply explained in multiple steps as follows. First, the process of identification of target agents including chemicals, biomolecules, and natural molecules by cell-based screening should be addressed [17]. Second, the selected target agents should be further evaluated by multiple EPC functional assays including cell proliferation, differentiation, and specific ability* in vitro* [18]. Third, the primed EPCs treated by target reagents should be confirmed in ischemic animal model mice by transplanting cultured cells into ischemic mouse model [19] and eventually clarified the* in vivo* molecular mechanism of the blood vessel repair [20]. Many studies focused on enhancing EPC functionalities by screening target intracellular signaling molecules indicate that appropriate control of intracellular antioxidant production promotes stem/progenitor cell bioactivities, including cell proliferation, differentiation of transplanted cells into mature vascular cells, and long-term cell survival in ischemic tissues in ischemic CVDs [21, 22].

In this review, we will discuss the basis and generation of ROS, its cellular signaling, current studies on drug-based therapeutics against ROS production, and cell-based therapeutics against oxidative stress-induced vascular diseases. In particular, we will discuss recent promising strategies that enhance EPC function by blocking excess ROS production, which induces blood vessels injury, to provide a novel direction for future cell-based therapies for blood vessel repair.

## 2. Reactive Oxygen Species

### 2.1. Basis of ROS

ROS are unavoidable by-products of aerobic metabolism [23]. ROS are generated at very high rates in organelles such as mitochondria [24], chloroplasts [25], and peroxisomes [26]. Uncommon chemical reactions during reduction and oxidation produce highly reactive oxygen compounds, including superoxide (O_2_^−^), peroxyl (RO_2_^−^), hydroxyl (OH^−^), and hydroperoxyl (HO_2_^−^) radicals and hydrogen peroxide (H_2_O_2_). ROS are harmful to cells because they damage lipids, proteins, and DNA. Lipid oxidation or peroxidation in erythrocytes causes hemolysis and carcinogenesis by affecting the oxidation process of proteins. This leads to protein fragmentation and protein-protein cross-linkages [27, 28]. In addition, increased intracellular ROS levels also induce cell damage.

### 2.2. Generation of ROS

In the ground state, an oxygen molecule contains two unpaired electrons. Addition of an electron fills one site of its two unpaired electrons, leading to the formation of ROS. Oxygen molecules form superoxide (O_2_^−^) anions [29], which are removed by superoxide dismutases (SODs). Conversion of excess superoxide anions by SODs is the key protection strategy in aerobic organisms. SODs convert superoxide anions into H_2_O_2_ [30]. Although H_2_O_2_ is not a powerful oxidizing agent, it can easily penetrate cells. H_2_O_2_ is removed by two enzymes, namely, catalase and peroxidase, by using different reductants such as ascorbate [31], glutathione [32], thioredoxin [33], phenolic compounds [34], and reduced nicotinamide adenine dinucleotides [35, 36] to produce oxygen (O_2_) and two hydrogen oxide (H_2_O) molecules.

H_2_O_2_ is nontoxic to cells; however, H_2_O_2_ combines with transition metals such as Fe^2+^ [37] and Cu^+^ [38] to form hydroxyl radicals (HO^−^) through fenton reaction. These hydroxyl radicals, which are highly reactive, react with almost every molecule, including DNA [39], membrane lipids [40], and carbohydrates [41], present in living cells to induce cellular dysfunction [42, 43].

### 2.3. Cellular Effects of ROS

Hydroxyl radicals react with organic compounds. These radicals bind to double bonds in heterocyclic DNA bases and attack double bonds in pyrimidines and purines at diffusion-controlled rates [44]. Electron density of target molecules in the reaction site is important for this reaction. Reaction of hydroxyl radicals with nucleotides produces various final products [45]. Substitution with mutated nucleotide bases in DNA results in base pair mismatch [46]. A single-point mutation can change the entire DNA sequence. Furthermore, effect of hydroxyl radicals on nucleotide bases induces single-and double-stranded DNA breaks [47–49].

Accumulation of DNA damage is closely associated with carcinogenesis [50, 51]. One example is mutations in tumor suppressor genes. Tumor suppressor genes encoding p53 [52] and Ras [53] show GC to TA transversions in lung and liver cancers [54, 55]. ROS-related signaling pathways are upregulated in various cancers [56]. H_2_O_2_ acts as a secondary messenger and regulates protein activity [57]. Excessive activation of tyrosine phosphatases, protein tyrosine kinases, receptor kinases, and transcription factors contributes to carcinogenesis [58].

On the other hands, ROS are essential for regulating different signaling pathways [59]. ROS induce reversible posttranslational protein modifications. H_2_O_2_ thiol groups on cysteine residues to produce sulfenic acid (-SOH) [60]. Sulfenic acid reacts with glutathione (GSH) [61] to become glutathionylated (-SSG) [62], with amides to form a sulfenyl amide (-SN-) [63] or with nearby thiols to form disulfide bonds (-SS-) [64, 65]. Thus, ROS modulate signaling pathways by modifying the activity of target proteins [66]. Furthermore, ROS oxidize and modify several proteins. ROS oxidize p53 [67], Jun [68], and Fos [69] to decrease their transcriptional activity. In contrast, ROS-induced oxidation of p50 [70] stimulates its transcriptional activity. Recent studies on stem cells indicate that mitochondria-targeted antioxidants or knockdown of the gene encoding complex III Rieske iron sulfur protein inhibits the differentiation of human mesenchymal stem cells (hMSCs) to adipocytes [71, 72]. Thus, ROS are referred to as “a double-edged sword.”

Various ROS are generated through enzymatic processes by intracellular enzymes including NADPH oxidase, xanthine oxidoreductase, nitric oxide synthase (NOS), and myeloperoxidase (MPO) and through nonenzymatic processes. NADPH oxidases are a family of multiple-subunit complex enzymes that use NADPH as an electron source. Seven nox isoforms containing two membrane bound subunits gp91 and p22 and several cytoplasmic subunits (G protein, p40, p47, and p67) have been identified [73]. Xanthine oxidoreductase is composed of xanthine oxidase (XO) and xanthine dehydrogenase in the same enzyme. Only XO generates superoxide anions and H_2_O_2_ [74]. NOS catalyze the reaction of L-arginine to L-citrulline with the production of nitric oxide (NO). Different isoforms of NOS include neuronal NOS (nNOS), endothelial NOS (eNOS), and inducible NOS (iNOS) [75]. MPO belongs to a family of heme peroxidases. ROS generated by this enzyme oxidize lipids and proteins [76].

## 3. Oxidative Stress-Related CVDs

Blood vessels are widely distributed in the body; they supply nutrients for cellular needs and remove unnecessary substances. As blood vessels throughout the entire body are connected, one type of CVD can easily induce a secondary disease. Unlike other by-products, ROS damage EC and vascular SMCs [77]. For example, ROS-induced disruption of NO balance induces vasorelaxation. Moreover, ROS induce various vascular diseases because of their strong reactivity [78].

The majority of vascular diseases result from atherosclerosis [79]. ROS from SMCs and ECs move toward artery walls and react with low-density lipoprotein (LDL) [80] to produce oxidized LDL (Ox-LDL), damaging ECs [81]. Ox-LDL induces the expression of chemotactic factors such as macrophage colony stimulating factor [82] which activate T lymphocytes and monocytes that attach to ECs [83]. Moreover, growth factors secreted by ECs promote the migration of monocytes into cell adhesion sites [84]. Monocytes and lipoproteins generate ROS, which convert Ox-LDL into highly oxidized LDL [85]. Moreover, macrophages engulf this highly oxidized LDL to become foam cells [86]. With leukocyte, formed cells convert into fatty streak, where migrated SMC and fatty streak mixture become fibrous, forming fibrous cap after calcification. Excessive formation of fibrous cap results in sudden rupture, to produce thrombi-like substances that close the blood vessel. These processes occur in various tissues [87, 88], including the heart during myocardial infarction [89], the brain during stroke [90], and the kidneys [91]. Another atherosclerosis induced disease is hypertension [92], which is also independently associated with ROS. Under normal conditions, eNOS catalyzes the production of NO from arginine [93]. Insufficient levels of tetrahydrobiopterin (H_4_B) or arginine switch eNOS from a coupled state, which generate NO, to an uncoupled state, which generate peroxides. Increased levels of vascular peroxides result in the oxidation of H_4_B, leading to additional NOS uncoupling. Decreased NO levels are not sufficient to relax SMCs, and this impaired relaxation of blood vessels is closely associated with hypertension [94].

## 4. Therapeutic Use of Antioxidants in CVDs

Many studies suggest that ROS are involved in the development of CVDs and play a causal role in atherothrombosis and other vascular diseases in various animal models. Some ROS inhibitors have been used for treating CVDs. The most relevant antioxidants that exert beneficial cardiovascular effects of ROS inhibitors are ascorbic acid (vitamin C), *α*-tocopherol (vitamin E), and *β*-carotene [95, 96]. Vitamin C reverses endothelial dysfunction in patients with coronary artery disease [97] and attenuates abnormal vasomotor reactivity [98]. However, large-scale randomized trials evaluating vitamin C indicate no effect on CVDs [99]. Similarly, large randomized trials have not shown substantial cardioprotective effects of these antioxidants. A meta-analysis of randomized controlled trials failed to show the cardioprotective effects of these antioxidants and observed that these antioxidants did not reduce the number of clinical events in high-risk patients or in patients with an established disease [100, 101].

Vitamin E is one of the most effective antioxidants for preventing CVDs because of its cardioprotective effects [102]. Vitamin E has four tocopherols and four tocotrienols. Each vitamin E type consists of four isoforms (alpha (*α*), beta (*β*), gamma (*γ*), and delta (*δ*)). Of the eight types of molecules, the activity of *α*-tocopherol defines the quality of vitamin E. Lipid-soluble antioxidant *α*-tocopherol reacts with lipid radicals and protects the cell membrane. *γ*-Tocopherol, another form of vitamin E, reacts with electrophilic mutagens and inhibits carcinogenesis [103, 104]. In addition, vitamin E not only decreases the risk of coronary heart disease [105–108], but also reduces coronary artery disease and cardiovascular events.

*β*-Carotene, a reddish-orange pigment found in certain fruits and vegetables, is type of carotene. This phytonutrient quenches singlet oxygen with greater efficiency [109]. *β*-Carotene is a precursor of vitamin A and is converted into bioactive retinol (the bioactive form of vitamin A) which prevents lipid oxidation of cellular membranes. It has a similar effect of vitamin E but utilizes a different mechanism. It also protects human LDL from copper-stimulated oxidation [110]. Several epidemiological studies have shown that *β*-carotene levels are associated with reduced risk of CVDs and heart attacks [111, 112].

Lack of cardioprotective effects of currently used antioxidants has resulted in the development of new and more effective ROS inhibitors. Although mitochondria play a pivotal role in the pathogenesis of CVDs, no study has been conducted to determine mitochondria-targeting ROS inhibitors in patients with CVDs. Antioxidants therapy targeting ROS production by mitochondria might be more effective than conventional antioxidant therapy for treating CVDs [113].

## 5. Cell-Based Therapeutic Strategies against Oxidative Stress-Related Diseases

The BM is a reservoir of hematopoietic stem cells (HSCs) and generates various hematopoietic cells. Therefore, multiple diseases, including leukemia, lymphoma, and immune deficiency, can be treated by performing BM transplantation [114]. However, this therapeutic approach requires healthy donor BM-derived HSCs for regenerating functional stem cells and hematopoietic lineage cells to replace damaged tissues. In addition, BM transplantation is strictly regulated, for avoiding graft versus host disease [115]. Allogeneic adult stem cell therapy was developed in 2012 to overcome the limitations associated with BM transplantation with BM-derived cells [116]. One approach is to isolate MSCs from the BM of adult donors and freeze these cells until further use; MSCs do not express MHC class I and rarely express MHC class II, thus allowing successful allogeneic transplantation by preventing immune rejection [117, 118]. Recently, multiple adult stem cells including neural stem cells (NSCs), HSCs, EPCs, and MSCs were intensively studied for use in stem cell-based therapy.

### 5.1. Adult Stem Cell-Based Therapy against ROS

Stem cell niche is a microenvironment in which adult stem cells interact with adjacent cells. In addition to cells, stem cell niche contains cytokines, growth factors, and oxygen as other important components. Stem cells strictly preserve multiple capabilities, including self-renewal, proliferation, and lineage differentiation, by communicating with these different factors [119]. Quiescence of HSCs can be controlled using an adequate redox regulation system for ROS for maintaining the stemness of these cells. Although ROS production is unavoidable during cellular metabolism, high levels stimulate HSC differentiation, senescence, and apoptosis [120, 121]. However,* ex vivo* cultured EPCs maintain appropriate ROS level to promote the repair of blood vessel injury [122, 123]. Therefore, maintenance of a healthy status of adult stem/progenitor cells in the presence of ROS both* in vitro* and* in vivo* can be used as a therapeutic strategy and should be addressed in detail by studying ROS biology.

### 5.2. Understanding ROS Signaling for Adult Stem Cell Biology

The role of ROS in adult stem cell biology has been intensively studied using MSCs, HSCs, and NSCs because these major types of adult stem cells have similar properties. First, these stem cells prefer aerobic glycolysis to oxidative phosphorylation as an energy source. Second, these stem cells maintain low ROS levels, thus preserving sufficient glycolytic metabolites. Third, these stem cells have low ATP/cell content and maintain high oxygen consumption rate [124]. However, precise signaling cascades induced by ROS seem to be slightly different.

MSCs can be derived from diverse sources, including the adipose tissue, BM, and dental pulp and can differentiate into various cell types [125]. Although MSCs are a good candidate for promising cell-based therapy against tissue injury, they are very sensitive to oxygen concentration in ischemic tissues or in* ex vivo* cultures. In MSCs, mitogen-activated protein kinases (MAPKs) such as c-Jun N-terminal kinases (JNKs), p38, and extracellular signal-regulated kinases (ERKs) are activated by ROS. This results in the activation of apoptotic proteins and suppression of antiapoptotic signaling pathways [126], which is one of many reasons why most MSCs do not survive after transplantation [127]. In* in vitro* cultures, ROS regulate MSC differentiation into three lineages, namely, adipocyte, osteocyte, and chondrocyte [128].

In HSCs, ROS generation is increased abnormally during hematopoietic homeostasis under pathological conditions [129]. Uncoordinated ROS production inhibits the self-renewal and induces the senescence of HSCs, resulting in hematopoietic dysfunction [130]. To address the importance of ROS in HSCs, ataxia telangiectasia mutated (ATM), DNA mutation, and DNA damage checkpoint pathway in transgenic mice were intensively examined because ATM knockout (ATM-KO) mice with an abnormal ROS status show impaired hematopoietic function with aging [131, 132]. Other groups have focused on the role of Foxo genes (*Foxo1*,* Foxo2*, and* Foxo3*) in ROS biology, because Foxo-KO mice also show hematopoietic dysfunctions similar to ATM-KO mice [133, 134]. In these mice, low ROS levels are required for HSCs proliferation, differentiation, and mobilization [135]. Accumulating evidences have clearly shown that hematopoietic reconstitution after HSC transplantation is strictly required for ROS-dependent proliferation of HSCs [136]. High ROS levels damage HSCs and affect MAPK and mammalian target of rapamycin (mTOR) signaling because exposure of mTOR-deficient HSCs to high ROS levels results in their sudden quiescence [137, 138].

Neuronal cells including neurons, astrocytes, microglia, and oligodendrocyte are generated from NSCs [139]. However, detailed mechanisms underlying ROS signaling pathways in NSCs are not completely understood. Neurogenic niche contains NSC and HSC [140]. These two types of adult stem cells show similar reaction to ROS, suggesting that NSC metabolism is similar to HSC. Some studies have shown that high endogenous ROS levels regulate proliferative NSC function including self-renewal and neurogenesis in PI3K/Akt-dependent manner [141].

### 5.3. ROS-Scavenging Chemicals in Stem Cells for Preclinical Study on CVDs

Muscle-derived stem cells preconditioned with* N*-acetylcysteine (an antioxidant) show significant increase in their survival ratio* in vivo* in a mouse model of myocardial infarction [142]. Another interesting chemical compound trimetazidine (TMZ) showed primed BM-MSCs with TMZ. Evaluation of BM-MSC survival after H_2_O_2_ treatment showed that preconditioned BM-MSCs were protected from H_2_O_2_ induced damage* in vitro*, because of the upregulation of hypoxia inducible factor-1alpha (HIF-1*α*), survivin, pAKT, and B-cell lymphoma 2 (Bcl-2). Evaluation of TMZ-preconditioned MSC function in an* in vivo* rat model of myocardial infraction clearly showed that transplantation of primed BM-MSCs significantly increased recovery capacity by activating pAKT and Bcl-2 expression [143], suggesting that appropriate modulation of ROS production enhances repair capabilities of cells transplanted in ischemic tissues.

## 6. Emerging Insights into Primed EPCs for Treating ROS-Related CVDs

Cell-based therapy is a promising strategy for treating patients with ROS-related CVDs [144]. Cell-based therapy involves improvement of stem cell function, including proliferation, differentiation, and antisenescence, by priming the cells with known chemical reagents or natural products. During the onset of severe CVDs, blood vessels injured by ROS form ischemic tissues. Emerging priming strategies for EPC based therapy against oxidative stress have intensively focused on restoring these ischemic tissues.

### 6.1. Protective Role of Lisosan G and Lady Joy against ROS in EPCs

Lisosan G (LG) is obtained from* Triticum sativum* (wheat). This grain is a dry powder and is registered with the Italian Ministry of Health as an alimentary integrator. Beans also contain large amounts of bioactive compounds [145]. Lady Joy (LJ) beans contain high levels of alpha-amylase inhibitor phaseolamin and genetically lack phytohemagglutinin (lectin), a toxic constituent [146]. Lucchesi et al. evaluated LG and LJ. EPCs were exposed to oxidative stress (H_2_O_2_) in the presence of LG and LJ [147]. These two compounds increase EPC viability and protect them against oxidative stress-induced damage. In addition, both LG and LJ improve the adhesion and decrease the senescence of EPCs. Furthermore, LG and LJ significantly decrease ROS generation in EPCs. To be specific, glutathione peroxidase-1 and superoxide dismutase-2 (SOD-2) are stimulated under the lysate with H_2_O_2_. They clearly showed that LG promoted Nrf-2 translocation into the nucleus, suggesting that LG and LJ protected EPC bioactivities in the presence of ROS.

### 6.2. Protective Role of Salvianolic Acid B against Oxidative Stress in EPCs

Radix salvia miltiorrhiza (also known as Tanshen in China) is a useful plant in eastern Asia, because of its perennial cultivation characteristic. In traditional Chinese medicine, it is used for treating chronic renal failure and coronary heart disease [148]. Salvianolic acid is another interesting natural product. Salvianolic acid has diverse isoforms and is associated with other polyphenolic acids [149, 150]. Tang et al. examined the protective effects of salvianolic acid B (SalB) against oxidative stress in BM-EPCs [149]. SalB-treated EPCs showed significantly increased migration ability and tube formation capacity, which did not affect their proliferation. SalB prevents H_2_O_2_ induced endothelial dysfunction by downregulating NOX4, eNOS, and NADPH oxidase. Furthermore, SalB inhibits caspase-3 activation and decreases Bax/Bcl-xL ratio after H_2_O_2_ treatment. In this study, they suggested that SalB-mediated angiogenesis required the activation of mTOR/p70S6K/4EBP1 pathway. They also showed that SalB downregulated MKK3/6-p38 MAPK-ATF2 and ERK1/2 pathways, suggesting that SalB protected EPCs from oxidative stress-induced damage.

### 6.3. Repair of ROS-Induced Blood Vessel Injury Using Fucoidan-Pretreated Senescent EPCs

Fucoidan, a sulfated polysaccharide, is extracted from various species of brown algae and seaweed [151]. This natural product has antiviral [152], antitumor [153], antithrombotic [154], anti-inflammatory [155], and antioxidant [156] properties. Fucoidan has an ionic structure, because of which it interacts with various angiogenic proteins [157], including basic fibroblast growth factor, to improve the proangiogenic properties of EPCs [158]. Lee et al. investigated the effects of fucoidan-preconditioning of EPCs both* in vitro* and* in vivo* in a mouse model of ischemia [159]. Treatment of senescent EPCs with fucoidan rescued the expression of functional surface markers CD34, c-Kit, VEGFR2, and CXCR4, and stimulated their tube formation ability* in vitro*. Furthermore, fucoidan stimulated the expression of cell cycle associated proteins Cdk4, cyclin D1, Cdk2, and cyclin E and enhanced FAK, Akt, and ERK pathways in senescent EPCs. Transplantation of fucoidan-preconditioned EPCs into ischemic tissues of a murine model of hindlimb ischemia repaired damaged blood vessels and markedly improved limb salvage.

### 6.4. Protective Role of Oleuropein against ROS in EPCs

Oleuropein (OLP) is present in olive oil extracted from olive leaves [160] and has high antioxidant activity [161]. OLP contains three subunits, namely, a glucose molecule, elenolic acid, and hydroxytyrosol [162].* In vitro* and* in vivo* studies indicate that OLP decreases the levels of superoxide anions and inhibits ROS production in leukocytes [163]. Choi et al. were the first to show the effect of OLP on vascular progenitor cells (VPCs) [164]. Angiotensin II significantly increases superoxide anion levels and decreases Prdx-1 and Prdx-2 levels in VPCs. OLP treatment significantly increased angiotensin II-induced decrease in Prdx-1 and Prdx-2 levels. These findings indicate that OLP decreases cellular ROS levels by regulating Prdx-1 and Prdx-2 expression and by activating ERK1/2 phosphorylation cascade, which is an upstream signal of Prdx-1 and Prdx-2. Thus, OLP stimulates the ERK1/2-Prdx pathway and reduces oxidative stress, thus enhancing Akt/eNOS signaling.

### 6.5. Protective Role of Tauroursodeoxycholic Acid against ROS in EPCs

Tauroursodeoxycholic acid (TUDCA) (also known as bile acid) is a taurine-conjugated ursodeoxycholic acid (UDCA). Bears contain large amount of TUDCA in their gall bladder. In Chinese medicine, ancient people used animal bile for several years. Moreover, TUDCA has been used for treating spasms and fevers. At present, TUDCA is used for treating of cholestatic liver disease [165]. TUDCA protects hepatocytes and restores glucose homeostasis by reducing ER stress. Cho et al. investigated the effect of TUDCA on blood vessel repair [166]. TUDCA treatment increases CD34^+^/Sca1^+^ progenitor cells in mouse peripheral blood and CD34^+^/CD31^+^/c-kit^+^ progenitor cells in human peripheral blood. Moreover, TUDCA promotes the differentiation of CD34^+^ HSCs into EPC lineage cells through the Akt signaling pathway. Increased expression of adhesion molecules on EPCs promotes their association with human aortic ECs. TUDCA treatment of a mouse model of hindlimb ischemia increased the populations of Flk-1^+^/CD34^+^ and Sca-1^+^/c-kit^+^ progenitor cells* in vivo*. Furthermore, c-kit^+^progenitor cells from a BM-transplanted model of hindlimb ischemia migrated to ischemic areas to repair damaged blood vessels. In addition, TUDCA significantly decreased p21 and p53 expression levels, which are associated with cellular senescence, increased NO levels, and decreased ROS levels. Transplantation of TUDCA-preconditioned senescent EPCs into ischemic tissues induced blood vessel regeneration.

## 7. Conclusion 

**Figure 1:**
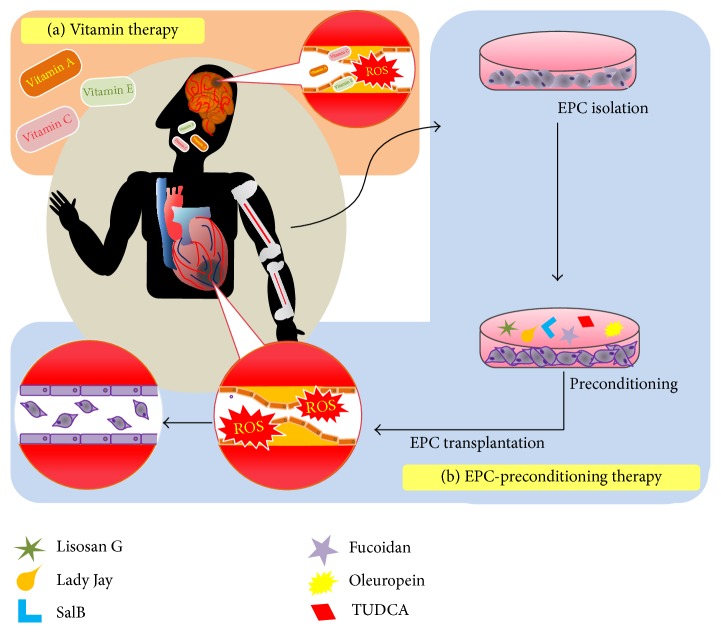
Schematic therapeutic strategies against cardiovascular diseases. (a) Vitamins therapy against ROS. (b) EPC-preconditioning therapy with antioxidant compounds. Transplanting preconditioned EPCs into ischemic tissues, such as myocardiac infarction and stroke.

Blood vessels are exposed to various harmful factors, including ROS, which trigger different secondary diseases. Particularly, ROS easily and highly react with other proteins in ECs and SMCs and induce multiple vascular diseases. Several research groups are attempting to overcome this pathological imbalance. A better understanding of oxidative stress signaling including ROS signaling will help in improving the bioactivities of adult stem cells, because excessive production of ROS negatively affects cellular senescence, proliferation, and differentiation. With respect to stem cell-based therapeutic strategies, several studies have clearly suggested that use of primed EPCs that block intracellular stress, including ROS, is a promising strategy for repairing oxidative stress-injured ischemic tissues. Precise understanding of drug-based therapeutics as ROS scavengers at molecular levels as well as priming of EPCs pretreated with ROS-scavenging chemicals or natural products will provide crucial and promising therapeutic approaches for treating oxidative stress-related CVDs (Figure 1).
